# Fold/Unfold Transformations for Fixpoint Logic

**DOI:** 10.1007/978-3-030-45237-7_12

**Published:** 2020-03-13

**Authors:** Naoki Kobayashi, Grigory Fedyukovich, Aarti Gupta

**Affiliations:** 8 Johannes Kepler University, Linz, Austria; 9 University of Birmingham, Birmingham, UK; 10 The University of Tokyo, Tokyo, Japan; 11 Florida State University, Tallahassee, USA; 12 Princeton University, Princeton, USA

## Abstract

Fixpoint logics have recently been drawing attention as common foundations for automated program verification. We formalize fold/unfold transformations for fixpoint logic formulas and show how they can be used to enhance a recent fixpoint-logic approach to automated program verification, including automated verification of relational and temporal properties. We have implemented the transformations in a tool and confirmed its effectiveness through experiments.

### 1 Introduction

A wide range of program properties can be verified by reducing to satisfiability/validity in a fixpoint logic [3–6, 18, 20, 22, 23, 29, 35]. In this paper, we build on top of `MuArith`, a first-order logic with least/greatest fixpoint operators and integer arithmetic, recently proposed by Kobayashi et al. [22]. It offers a powerful tool to handle the full class of modal $\mu$-calculus properties of while-programs (imperative programs with loops but without general recursion). In contrast, earlier studies on temporal program verification require different methods for each subclass of the modal $\mu$-calculus properties, such as LTL [12,16,28], CTL [2,3,13,34], and CTL$^*$ [11]. The recent program verifier based on `MuArith` [22] is effective in practice, i.e., by exploiting general-purpose solvers for Satisfiability Modulo Theories (SMT) and Constrained Horn Clauses (CHC), it can outperform tools designed specifically for CTL verification of C programs [13].

Despite these promising results, the generality of the fixpoint logic approach come at a cost. Since fixpoint logic formulas obtained by reduction from various verification problems often involve nested fixpoint operators, it could be challenging to check the validity of these formulas automatically. To enhance the capability of fixpoint logic provers, in this paper, we propose novel fold/unfold transformations and prove their correctness. These transformations are generally used to simplify relational verification, and in particular, to reduce the number of recurrences used in the program (or a set of programs) under analysis. Originally proposed for logic programming [8, 19, 32], they have been recently adopted for determining the satisfiability of CHC [15, 26] and allow discovery of *relational* invariants for a pair of loopy (or recursive) programs, as opposed to invariants within each individual program. Our transformations can be regarded as extensions of such transformations for a fixpoint logic, where quantifiers and arbitrarily nested least/greatest fixpoint operators are allowed.

We also present a procedure that seeks a way to apply the proposed fold/unfold transformations efficiently. Besides non-determinism in the choice of which fixpoint formulas to unfold, our "fold" operation replaces a formula $\phi$ with $P$ (where $P$ is the predicate defined by $P \stackrel{\triangle}{=} \phi$) and requires various reasoning to convert the current goal formula to a form $E[\phi]$, where the form of $E$ can be more complex than in the case of fold/unfold transformations for logic programming or CHC.

We have implemented the transformations and integrated them with the program verifier `Mu2CHC` [22] based on `MuArith`. We considered a number of examples of `MuArith` formulas which include formulas obtained from program verification problems for checking relational and temporal properties. Our new transformations allowed `Mu2CHC` to solve these formulas, which would not be doable otherwise.

To sum up, our contributions are: (i) a formalization of fold/unfold transformations for a fixpoint logic and proofs of their soundness, (ii) demonstration of the usefulness of the proposed transformations for verification of relational and temporal properties of programs, and (iii) a concrete procedure for automated transformation and its implementation and experiments.

The rest of this paper is structured as follows. Section 2 reviews the definition of the first-order fixpoint logic `MuArith` [22], and reductions from program verification problems to validity checking in `MuArith`. Section 3 formalizes our transformations and proves their correctness. Section 4 shows applications of our transformations to verification of relational and temporal properties of recursive programs. Section 5 reports an implementation and experimental results. Section 6 discusses related work and Section 7 concludes the paper.

### 2 First-Order Fixpoint Logic `MuArith`

We review the first-order fixpoint logic `MuArith` [22] in this section. `MuArith` is a variation of Mu-Arithmetic studied by Lubarsky [25] and Bradfield [7], obtained by replacing natural numbers with integers.

#### 2.1 Syntax

The set of (propositional) formulas, ranged over by $\varphi$, is defined in the following grammar.

$$\begin{aligned}
\varphi \text{ (formulas)} &::= a_1 \geq a_2 \mid P^{(k)}(a_1, \ldots, a_k) \mid \\
&\qquad \varphi_1 \vee \varphi_2 \mid \varphi_1 \wedge \varphi_2 \mid \forall x.\varphi \mid \exists x.\varphi \\
P^{(k)} \text{ ($k$-ary predicates)} &::= X^{(k)} \mid \lambda(x_1, \ldots, x_k).\varphi \mid \\
&\qquad \mu X^{(k)}(x_1, \ldots, x_k).\varphi \mid \nu X^{(k)}(x_1, \ldots, x_k).\varphi \\
a \text{ (arithmetic expressions)} &::= n \mid x \mid a_1 + a_2 \mid a_1 - a_2
\end{aligned}$$

The metavariable $\varphi$ represents a proposition, and $P$ denotes a predicate on (a tuple of) integers. We write $\top$ for $0 \geq 0$ and $\bot$ for $0 \geq 1$. In examples, we may also use other relational symbols such as $>$ and $=$. The meta-variable $x$ denotes an integer, and the meta-variable $X^{(k)}$ denotes a $k$-ary first-order predicate variable. We write $\mathtt{ar}(X^{(k)})$ for the arity of the predicate variable $X^{(k)}$, i.e., $k$; we often omit the superscript $(k)$ and just write $X$ for a predicate variable. The predicate $\mu X^{(k)}(x_1,\dots,x_k).\varphi$ (resp. $\nu X^{(k)}(x_1,\dots,x_k).\varphi$) denotes the least (resp. greatest) predicate $X$ such that $X(x_1,\dots,x_k)$ equals $\varphi$.

*Example 1.* Let $\mu X(x).(x = 0 \vee X(x-1))$ denote the least predicate $X$, such that $X(x) \equiv x = 0 \vee X(x-1) \equiv x = 0 \vee x-1 = 0 \vee X(x-2) \equiv \cdots$, i.e., $\lambda(x).x \geq 0$. In contrast, $\nu X(x).(x = 0 \vee X(x-1))$ denotes $\lambda(x).\top$. □

We write $\mathbf{FV}(\varphi)$ for the set of free (predicate and integer) variables in $\varphi$; $\forall x, \exists x, \mu X^{(k)}, \nu X^{(k)}$, and $\lambda x$ are binders. We sometimes write $\widetilde{x}$ for a sequence of variables $x_1,\dots,x_k$. We often write $\overline{\varphi}$ and $\overline{X}$ for the De Morgan dual of a formula $\varphi$ and a predicate variable $X$, respectively. For example, $\overline{\mu X(x).x = 0 \vee X(x-1)} = \nu\overline{X}(x).x \neq 0 \wedge \overline{X}(x-1)$. Here, $\overline{X}$ is a predicate variable, so the righthand side is $\alpha$-equivalent to $\nu X(x).x \neq 0 \wedge X(x-1)$. The overline for $X$ is used to indicate that it corresponds to the dual of $X$ in the original formula $\mu X(x).x = 0 \vee X(x-1)$.

### 2.2 Semantics

In this subsection, we define the formal semantics of formulas. Let $\mathbf{Z}$ be the set of integers, and $\mathbf{B} = \{\top_{\mathbf{B}}, \bot_{\mathbf{B}}\}$, with $\bot_{\mathbf{B}} \sqsubseteq_{\mathbf{B}} \top_{\mathbf{B}}$. Let $\mathbf{D}_k$ be the set $\mathbf{Z}^k \to \mathbf{B}$ of functions (where $\mathbf{Z}^k$ denotes the set of tuples consisting of $k$ integers). We define the partial order $\sqsubseteq_k$ on $\mathbf{D}_k$ by:

$$f \sqsubseteq_k g \Leftrightarrow \forall n_1,\dots,n_k \in \mathbf{Z}.f(n_1,\dots,n_k) \sqsubseteq_{\mathbf{B}} g(n_1,\dots,n_k).$$

Note that $(\mathbf{D}_k, \sqsubseteq_k)$ is a complete lattice, with $\lambda x_1.\cdots\lambda x_k.\bot_{\mathbf{B}}$ and $\lambda x_1.\cdots\lambda x_k.\top_{\mathbf{B}}$ as the least and greatest elements. We write $\bot_k$ and $\top_k$ for $\lambda x_1.\cdots\lambda x_k.\bot_{\mathbf{B}}$ and $\lambda x_1.\cdots\lambda x_k.\top_{\mathbf{B}}$, respectively. We also write $\sqcap^{(k)}$ (resp., $\sqcup^{(k)}$) for the greatest lower (resp., least upper) bound with respect to $\sqsubseteq_k$. We often omit $k$ and $\mathbf{B}$ and just write $\top, \bot, \sqsubseteq, \sqcap, \sqcup$, etc.. We often identify $\mathbf{B}$ and $\mathbf{D}_0 = \mathbf{Z}^0 \to \mathbf{B}$. We write $\mathbf{D}_k \to \mathbf{D}_\ell$ for the set of monotonic functions from $\mathbf{D}_k$ to $\mathbf{D}_\ell$.

We write $\mathbf{Env}$ for the set of functions that map each integer variable to an integer, and each $k$-ary predicate variable to an element of $\mathbf{D}_k$. For a formula $\varphi$ (resp., a predicate $P$ and an expression $a$) and an environment $\rho \in \mathbf{Env}$ such that $\mathbf{FV}(\varphi) \subseteq dom(\mathbf{Env})$ (resp., $\mathbf{FV}(P) \subseteq dom(\mathbf{Env})$ and $\mathbf{FV}(a) \subseteq dom(\mathbf{Env})$), Fig. 1 defines the semantics $[\![\cdot]\!]\rho$ of $\varphi$ (resp., $P$ and $a$), where for a monotonic function $F \in \mathbf{D}_k \to \mathbf{D}_k$, $\mathbf{LFP}^{(k)}(F) = \bigsqcap^{(k)}\{f \in \mathbf{D}_k \mid f \sqsupseteq_k F(f)\}$ and $\mathbf{GFP}^{(k)}(F) = \bigsqcup^{(k)}\{f \in \mathbf{D}_k \mid f \sqsubseteq_k F(f)\}$. When $\varphi$ and $P$ are closed (i.e., do not contain free variables), we just write $[\![\varphi]\!]$ and $[\![P]\!]$ for $[\![\varphi]\!]\emptyset$ and $[\![P]\!]\emptyset$ respectively. By abuse of notation, we often write $\varphi \sqsupseteq \psi$ if $[\![\varphi]\!]\rho \sqsupseteq [\![\psi]\!]\rho$ for any (valid) environment $\rho$ such that $\mathbf{FV}(\varphi) \cup \mathbf{FV}(\psi) \subseteq dom(\rho)$, and $\varphi \equiv \psi$ if $[\![\varphi]\!]\rho = [\![\psi]\!]\rho$; similarly for predicates. For example, $\exists z.(x > z \wedge z > y) \equiv (x > y+1) \sqsupseteq (x > y+2)$.

$$
\begin{aligned}
[\![a_1 \geq a_2]\!]\rho &= \begin{cases} \top \text{ if } [\![a_1]\!]\rho \geq [\![a_2]\!]\rho \\ \bot \text{ if } [\![a_1]\!]\rho < [\![a_2]\!]\rho \end{cases} \\
[\![P(a_1,\dots,a_k)]\!]\rho &= [\![P]\!]\rho([\![a_1]\!]\rho,\dots,[\![a_k]\!]\rho) \\
[\![X]\!]\rho &= \rho(X) \\
[\![\varphi_1 \vee \varphi_2]\!]\rho &= [\![\varphi_1]\!]\rho \sqcup [\![\varphi_2]\!]\rho \\
[\![\varphi_1 \wedge \varphi_2]\!]\rho &= [\![\varphi_1]\!]\rho \sqcap [\![\varphi_2]\!]\rho \\
[\![\forall x.\varphi]\!]\rho &= \textstyle\bigsqcap_{n\in\mathbf{Z}} [\![\varphi]\!]\rho\{x \mapsto n\} \\
[\![\exists x.\varphi]\!]\rho &= \textstyle\bigsqcup_{n\in\mathbf{Z}} [\![\varphi]\!]\rho\{x \mapsto n\} \\
[\![\lambda(x_1,\dots,x_k).\varphi]\!]\rho &= \lambda(n_1,\dots,n_k) \in \mathbf{Z}^k.[\![\varphi]\!]\rho\{x_1 \mapsto n_1,\dots,x_k \mapsto n_k\} \\
[\![\mu X^{(k)}(x_1,\dots,x_k).\varphi]\!]\rho &= \\
&\mathbf{LFP}^{(k)}(\lambda f \in \mathbf{D}_k.\lambda(n_1,\dots,n_k).[\![\varphi]\!]\rho\{X \mapsto f, x_1 \mapsto n_1,\dots,x_k \mapsto n_k\}) \\
[\![\nu X^{(k)}(x_1,\dots,x_k).\varphi]\!]\rho &= \\
&\mathbf{GFP}^{(k)}(\lambda f \in \mathbf{D}_k.\lambda(n_1,\dots,n_k).[\![\varphi]\!]\rho\{X \mapsto f, x_1 \mapsto n_1,\dots,x_k \mapsto n_k\}) \\
[\![n]\!]\rho &= n \\
[\![x]\!]\rho &= \rho(x) \\
[\![a_1 + a_2]\!]\rho &= [\![a_1]\!]\rho + [\![a_2]\!]\rho \\
[\![a_1 - a_2]\!]\rho &= [\![a_1]\!]\rho - [\![a_2]\!]\rho
\end{aligned}
$$

**Fig. 1.** The semantics of formulas.

*Example 2.* Recall formula $\mu X(x).x = 0 \vee X(x-1)$ from Example 1. We have $[\![\mu X(x).x = 0 \vee X(x-1)]\!]\emptyset = \mathbf{LFP}^{(1)}(F)$, with $F = \lambda f \in D_1.\lambda n \in \mathbf{Z}.(n = 0) \sqcup f(n-1)$. Since for any $m$, $F^m(\lambda x \in \mathbf{Z}.\bot) = \lambda n \in \mathbf{Z}.0 \leq n \leq m-1$, we have $\mathbf{LFP}^{(1)}(F) = \lambda n \in \mathbf{Z}.0 \leq n$ (here, $\leq$ denotes the semantic relation on integers). In contrast, $[\![\nu X(x).x = 0 \vee X(x-1)]\!]\emptyset = \mathbf{GFP}^{(1)}(F) = \lambda n \in \mathbf{Z}.\top$. □

#### 2.3 Program Verification as Validity Checking of `MuArith` Formulas

Various verification problems for first-order recursive programs can be reduced to validity of `MuArith` formulas. We refer the reader to [22] for a general reduction schema from temporal properties to `MuArith` formulas. However, as shown in this subsection, some formulas require additional handling that motivates the need for new transformations to be presented in Section 3.

Consider the following functional program (written in the syntax of OCaml) that multiplies two numbers.

```
let rec mult(x, y) = if y=0 then 0 else x + mult(x,y-1)
```

Then, the ternary relation $Mult(x, y, r)$ that expresses "`mult`$(x, y)$ terminates and returns $r$" is expressed as the following `MuArith` formula:

$$\mu Mult(x, y, r).(y = 0 \wedge r = 0) \vee \exists s.(y \neq 0 \wedge r = x + s \wedge Mult(x, y-1, s)).$$

This lets us express a partial correctness property "if $P(x, y)$ holds and $\texttt{mult}(x, y)$ terminates and returns $r$, then $Q(x, y, r)$ holds" by: $\forall x, y, r. P(x, y) \wedge \mathit{Mult}(x, y, r) \Rightarrow Q(x, y, r)$. It can further be rewritten to the following `MuArith` formula:

$$\forall x, y, r. \overline{P}(x, y) \vee \overline{\mathit{Mult}}(x, y, r) \vee Q(x, y, r), \tag{1}$$

where $\overline{P}$ and $\overline{\mathit{Mult}}$ are respectively De Morgan duals of $P$ and $\mathit{Mult}$; $\overline{\mathit{Mult}}$ can be expressed by:

$$\nu \overline{\mathit{Mult}}(x, y, r).(y \neq 0 \vee r \neq 0) \wedge \forall s.(y = 0 \vee r \neq x + s \vee \overline{\mathit{Mult}}(x, y - 1, s)).$$

The total correctness "if $P(x, y)$, then $\texttt{mult}(x, y)$ terminates and returns $r$, such that $Q(x, y, r)$" can be expressed by: $\forall x, y. P(x, y) \Rightarrow \exists r. \mathit{Mult}(x, y, r) \wedge Q(x, y, r)$, which is equivalent to the `MuArith` formula:

$$\forall x, y. \overline{P}(x, y) \vee (\exists r. \mathit{Mult}(x, y, r) \wedge Q(x, y, r))$$

As a special case, the termination property "if $y \geq 0$ then $\texttt{mult}(x, y)$ terminates" can be expressed by:

$$\forall x, y. y < 0 \vee \exists r. \mathit{Mult}(x, y, r). \tag{2}$$

We can also express relational properties of programs such as the equivalence of two programs. Let us consider another implementation of multiplication:

```
let mult2(x,y) =
  let rec multacc(x,y,a) = if y=0 then a else multacc(x,y-1,x+a)
in multacc(x,y,0)
```

Then predicate $\mathit{Multacc}(x, y, a, r)$ which represents "$\texttt{multacc}(x, y, a)$ terminates and returns $r$" can be expressed by:

$$\mu \mathit{Multacc}(x, y, a, r).(y = 0 \wedge r = a) \vee (y \neq 0 \wedge \mathit{Multacc}(x, y - 1, x + a, r)).$$

Thus, the equivalence of `mult` and `mult2` can be expressed by: $\forall x, y, r. \mathit{Mult}(x, y, r) \Leftrightarrow \mathit{Multacc}(x, y, 0, r)$, which can be expressed by the conjunction of the `MuArith` formulas:

$$\forall x, y, r. \overline{\mathit{Mult}}(x, y, r) \vee \mathit{Multacc}(x, y, 0, r) \tag{3}$$

$$\forall x, y, r. \mathit{Mult}(x, y, r) \vee \overline{\mathit{Multacc}}(x, y, 0, r) \tag{4}$$

where $\overline{\mathit{Multacc}}$ is the De Morgan dual of $\mathit{Multacc}$, defined analogously to $\overline{\mathit{Mult}}$.

**Motivation.** Kobayashi et al. [22] presented a method for proving the validity of `MuArith` formulas. It can prove formula (1) valid: since there are neither $\mu$ nor $\exists$, it is reducible to the problem of satisfiability of CHC [4]. However, the method is not powerful enough on formulas (2) and (3) for termination and program equivalence, respectively. It first tries to eliminate existential quantifiers and $\mu$-formulas, so that the resulting formula can be reduced to the satisfiability of

CHC. But it fails when the witness of an existential quantifier (i.e., $r$ such that $\exists r.\varphi$) is not bounded by a linear expression, e.g., the witness for $\exists r$ is a non-linear expression $x \times y$ in the case of (2). This is unfortunate, as methods specialized on proving program termination, e.g. [18], can easily prove the termination of program `mult`. Thus, in order to exploit the advantage of the uniform approach to program verification based on `MuArith`, we need to strengthen the method for proving `MuArith` formulas.

#### 2.4 Auxiliary Definitions

We introduce additional definitions on formulas, which will be used later in our formalization of fold/unfold-like transformations. A $(k, \ell)$-*context* (or, just a context) is an expression obtained from an $\ell$-ary predicate by replacing a $k$-ary predicate variable with $[\,]$ (in other words, a context is a predicate that may contain $[\,]$ as a special predicate variable). For a context $C$ and a predicate $P$ (that does not contain free occurrences of variables bound in $C$), we write $C[P]$ for the predicate obtained by replacing $[\,]$ with $P$. For example, $C \triangleq \lambda(x, y).\exists z.[\,](x, z, y)$ is a $(3, 2)$-context, and $C[\lambda(x, y, z).(x > y \wedge y > z)]$ is $\lambda(x, y).\exists z.(\lambda(x, y, z).(x > y \wedge y > z))(x, z, y)$, which is equivalent to $\lambda(x, y).\exists z.x > z \wedge z > y$.

For a function $F \in \mathbf{D}_k \to \mathbf{D}_\ell$, we say that $F$ is *continuous* if it preserves the least upper bound, i.e., $F(\bigsqcup_{f \in S} f) = \bigsqcup_{f \in S} F(f)$ for any (possibly infinite) set $S \subseteq \mathbf{D}_k$. Similarly, we say that $F$ is *co-continuous* if it preserves the greatest lower bound, i.e., $F(\bigsqcap_{f \in S} f) = \bigsqcap_{f \in S} F(f)$. For example, $\lambda f.f \wedge g \in \mathbf{D}_0 \to \mathbf{D}_0$ and $\lambda f.f \wedge g$ is both continuous and co-continuous for any $\varphi \in \mathbf{D}_0$. In contrast, $\lambda f.\exists x.f(x) \in \mathbf{D}_1 \to \mathbf{D}_0$ is continuous but not co-continuous;[4] $\lambda f.\forall x.f(x) \in \mathbf{D}_1 \to \mathbf{D}_0$ is co-continuous but not continuous. We say that a context $C$ is continuous if its semantics, i.e., $\lambda f.[\![C[X]]\!]\{X \mapsto f\}$ is; analogously for co-continuity.

The following lemma (which follows immediately from the definition) provides a syntactic condition that is sufficient for the co-continuity of a context.

**Lemma 1.** *Let $C$ be a $(k, \ell)$-context. If $C$ can be generated by the following syntax, then $C$ is co-continuous.*[5]

$$C ::= [\,] \mid \lambda(x_1, \dots, x_k).C \mid C(a_1, \dots, a_k) \mid C \wedge \varphi \mid \varphi \wedge C \mid C \vee \varphi \mid \varphi \vee C \mid \forall x.C$$

*Remark 1.* The syntax and semantics of `MuArith` was defined based on hierarchical fixpoint equations (HES) in [22]. The above semantics is equivalent to that of [22], modulo the standard conversions between fixpoint formulas and HES.

[4] In fact, let $F = \lambda f.\exists x.f(x) \in \mathbf{D}_1 \to \mathbf{D}_0$ and $S = \{\lambda x.x \geq n \mid n \in \mathbf{Z}\}$. Then $F(f) = \top$ for any $f \in S$, but $F(\bigsqcap_{f \in S} f) = F(\lambda x.\bot) = \bot$.

[5] Here, for the sake of simplicity, we mix the syntax of contexts that yield predicates and propositions.

## 3 Fold/Unfold-Like Transformations

In this section, we present new fold/unfold-like transformations for `MuArith`, to enhance the power of `MuArith` validity checkers. We first informally review fold/unfold transformations for logic programming and explain what kind of transformation we wish to apply to `MuArith` formulas in Section 3.1. We then prove theorems that justify such transformations in Sections 3.2 and 3.3.

### 3.1 Overview of Transformations for `MuArith`

**Revisiting Fold/Unfold Transformations for Logic Programming** The original concept [32] is presented in the following example, where each recurrence is represented by a CHC (i.e., an implication involving uninterpreted predicates *Even* and *Odd*).

$$\begin{array}{ll} \mathit{Even}(x) \Leftarrow x = 0 & \mathit{Even}(x) \Leftarrow x > 0, \mathit{Even}(x-2) \\ \mathit{Odd}(x) \Leftarrow x = 1 & \mathit{Odd}(x) \Leftarrow x > 0, \mathit{Odd}(x-2) \end{array}$$

We wish to prove that $\bot \Leftarrow \mathit{Even}(x), \mathit{Odd}(x)$. Many of the existing CHC solvers, such as `HoICE` [9] and `Z3` [24], fail to prove it as they do not handle the divisibility constraints well. After defining a new predicate *EvenOdd* as $\mathit{EvenOdd}(x) \Leftarrow \mathit{Even}(x), \mathit{Odd}(x)$ and unfolding *Even*, we obtain the following new CHCs.

$$\mathit{EvenOdd}(x) \Leftarrow x = 0, \mathit{Odd}(x) \qquad \mathit{EvenOdd}(x) \Leftarrow x > 0, \mathit{Even}(x-2), \mathit{Odd}(x)$$

By unfolding $\mathit{Odd}(x)$ in the first CHC, its body becomes inconsistent. By unfolding $\mathit{Odd}(x)$ in the second CHC, we obtain the following new CHCs.

$$\begin{array}{l} \mathit{EvenOdd}(x) \Leftarrow x > 0, \mathit{Even}(x-2), x = 1 \\ \mathit{EvenOdd}(x) \Leftarrow x > 0, \mathit{Even}(x-2), \mathit{Odd}(x-2) \end{array}$$

By unfolding $\mathit{Even}(x-2)$, the body of the first CHC becomes inconsistent. Now, the part "$\mathit{Odd}(x-2), \mathit{Even}(x-2)$" in the second CHC matches the definition of *EvenOdd*, so we can "fold" it and obtain the following new CHC.

$$\mathit{EvenOdd}(x) \Leftarrow x > 0, \mathit{EvenOdd}(x-2)$$

The least solution for *EvenOdd* is $\lambda x.\bot$, hence we have now obtained $\bot \Leftarrow \mathit{Even}(x), \mathit{Odd}(x)$ without synthesizing interpretations of *Even* and *Odd* over the divisibility constraints.

**Transformations for `MuArith`.** The above example can be reformulated in `MuArith`. Predicates *Even* and *Odd* are expressed as follows.

$$\mu \mathit{Even}(x).x = 0 \vee (x > 0 \wedge \mathit{Even}(x-2)) \tag{5}$$

$$\mu \mathit{Odd}(x).x = 1 \vee (x > 0 \wedge \mathit{Odd}(x-2)) \tag{6}$$

We wish to prove that $Even(x) \wedge Odd(x)$ is inconsistent, i.e. $\forall x.\overline{Even}(x) \vee \overline{Odd}(x)$ is valid where $\overline{Even}$ and $\overline{Odd}$ are:

$$\nu\overline{Even}(x).x \neq 0 \wedge (x \leq 0 \vee \overline{Even}(x-2)) \tag{7}$$

$$\nu\overline{Odd}(x).x \neq 1 \wedge (x \leq 0 \vee \overline{Odd}(x-2)) \tag{8}$$

Now, let $Y(x) \stackrel{\triangle}{=} \overline{Even}(x) \vee \overline{Odd}(x)$, which can be rewritten as follows.

$$\begin{aligned} Y(x) &\equiv (x \neq 0 \wedge (x \leq 0 \vee \overline{Even}(x-2))) \vee (x \neq 1 \wedge (x \leq 0 \vee \overline{Odd}(x-2))) \\ &\equiv (x \leq 0 \vee x \neq 1 \vee \overline{Even}(x-2))) \wedge (x \leq 0 \vee \overline{Even}(x-2) \vee \overline{Odd}(x-2)) \\ &\equiv x \leq 0 \vee \overline{Even}(x-2) \vee \overline{Odd}(x-2) \equiv x \leq 0 \vee Y(x-2) \end{aligned}$$

Based on this, we wish to replace $Y$ with $\nu Y(x).x \leq 0 \vee Y(x-2)$; then the validity of $\forall x.Y(x)$ would follow immediately. As we will see later in Section 3.3, this transformation is indeed sound.

Intuitively, the above transformation works as follows. Given a formula $C[X]$, which contains a fixpoint formula $X$ defined by the equation $X = D[X]$, introduce a new predicate $Y$, such that $Y = C[X]$. Then, unfold $X$ to $D[X]$ and obtain $Y = C[D[X]]$. Then, rewrite $C[D[X]]$ to a formula of the form $E[C[X]]$. By "folding" $C[X]$, we obtain $Y = E[Y]$, which serves as a new definition clause for $Y$. We wish to apply this kind of transformation not only to $\nu$-only formulas like above, but also to formulas involving $\mu$ and quantifiers, as discussed below.

Recall formula (2) from Section 2.3. Let $X(x,y) \stackrel{\triangle}{=} \exists r.Mult(x,y,r)$. Then,

$$\begin{aligned} X(x,y) &\equiv \exists r.((y = 0 \wedge r = 0) \vee \exists s.(y \neq 0 \wedge r = x + s \wedge Mult(x, y-1, s))) \\ &\equiv y = 0 \vee (y \neq 0 \wedge \exists s.Mult(x, y-1, s)) \\ &\equiv y = 0 \vee (y \neq 0 \wedge X(x, y-1)). \end{aligned}$$

As justified later in Section 3.2, we can then replace $X$ with $\mu X(x,y).y = 0 \vee (y \neq 0 \wedge X(x, y-1))$. We are then left with formula $\forall x, y.y < 0 \vee X(x,y)$, which can then be proved valid by `Mu2CHC` [22], the existing `MuArith` validity checker.

Let us also recall a generalized version of formula (3):

$$\forall x, y, a, r.\overline{Mult}(x,y,r) \vee Multacc(x,y,a,r+a),$$

which contains $\mu$ and $\nu$. Let $Y(x,y,a,r) \stackrel{\triangle}{=} \overline{Mult}(x,y,r) \vee Multacc(x,y,a,r+a)$. Then, we have:

$$\begin{aligned} Y(x,y,a,r) \equiv\ & ((y \neq 0 \vee r \neq 0) \wedge \forall s.(y = 0 \vee r \neq x + s \vee \overline{Mult}(x, y-1, s))) \\ & \vee (y = 0 \wedge r + a = a) \vee (y \neq 0 \wedge Multacc(x, y-1, x+a, r+a)) \\ \equiv\ & (y = 0 \Rightarrow r \neq 0 \vee r + a = a) \\ & \wedge (y \neq 0 \Rightarrow (\overline{Mult}(x, y-1, r-x) \vee Multacc(x, y-1, x+a, r+a))) \\ \equiv\ & y \neq 0 \Rightarrow Y(x, y-1, x+a, r-x) \end{aligned}$$

As justified in Section 3.3, we can replace $Y$ with $\nu Y(x,y,a,r).(y = 0 \vee Y(x, y-1, x+a, r-x))$, giving us $\forall x, y, a, r.Y(x,y,a,r)$ immediately.

Although the above transformations are sound, the soundness of fold/unfold transformations for `MuArith` is delicate in general. For example, consider formula $\exists x. x \geq y \wedge X(x, y)$, where:

$$X \stackrel{\triangle}{=} \nu X(x, y). x \geq y + 1 \wedge X(x, y + 1).$$

It is obviously false since there exists no $x$ that satisfies $x \geq y \wedge x \geq y + 1 \wedge x \geq y + 2 \wedge \cdots \equiv \forall n \geq 0. x \geq y + n$. Let $Y(y) \stackrel{\triangle}{=} \exists x. x \geq y \wedge X(x, y)$. Then,

$$\begin{aligned} Y(y) &\equiv \exists x.(x \geq y \wedge x \geq y + 1 \wedge X(x, y + 1)) \\ &\equiv \exists x.(x \geq y + 1 \wedge X(x, y + 1)) \equiv Y(y + 1). \end{aligned}$$

Based on this, one may be tempted to replace $Y$ with $\nu Y(y). Y(y + 1) \equiv \lambda y. \top$, but that is obviously wrong.

In the next two subsections, we present theorems that justify all the transformations above except the last (invalid) one.

#### 3.2 Transformations for $\mu$-Formulas

In this subsection, we prove a theorem that enables the replacement of a predicate of the form $C[\mu X. D[X]]$ with one of the form $\mu Y. E[Y]$ and applies it to justify the transformation for $\exists r. Mult(x, y, r)$ discussed in the previous subsection. The corresponding transformation for $\nu$-formulas is discussed in the next subsection. The theorem is stated as follows.

**Theorem 1.** *Let $C, D$ and $E$ be $(k, \ell)$, $(k, k)$, and $(\ell, \ell)$-contexts respectively. If $C[D[X]] \sqsupseteq_\ell E[C[X]]$ holds for any $k$-ary predicate $X$, then we have:*

$$C[\mu X(x_1, \ldots, x_k). D[X](x_1, \ldots, x_k)] \sqsupseteq_\ell \mu Y(y_1, \ldots, y_\ell). E[Y](y_1, \ldots, y_\ell).$$

The theorem follows easily from the definition of the semantics of the least fixpoint operator.

*Proof.* Suppose $C[D[X]] \sqsupseteq E[C[X]]$. Then, we have

$$C[\mu X(\widetilde{x}). D[X](\widetilde{x})] \equiv C[D[\mu X(\widetilde{x}). D[X](\widetilde{x})]] \sqsupseteq E[C[\mu X(\widetilde{x}). D[X](\widetilde{x})]].$$

Since $\mu Y(\widetilde{y}). E[Y](\widetilde{y})$ is the least predicate $Y$ such that $Y \sqsupseteq E[Y]$, we have $C[\mu X(\widetilde{x}). D[X](\widetilde{x})] \sqsupseteq \mu Y(\widetilde{y}). E[Y](\widetilde{y})$ as required. □

To see how the theorem above enables fold/unfold-like transformations, suppose that we wish to prove a formula of the form $Y \equiv C[\mu X(\widetilde{x}). D[X](\widetilde{x})]$. It suffices to prove $C[D[\mu X(\widetilde{x}). D[X](\widetilde{x})]]$, obtained by unfolding $X$. If the assumption $C[D[X]] \sqsupseteq E[C[X]]$ holds, we can change the goal to $E[C[\mu X(\widetilde{x}). D[X](\widetilde{x})]]$. Thus, by the theorem, it suffices to prove $\mu Y(\widetilde{y}). E[Y](\widetilde{y})$, which is obtained by "folding" $C[\mu X. D[X](\widetilde{x})]$ to $Y$. Note that the theorem guarantees only that the transformation provides an *underapproximation* of the original predicate. A stronger condition is required for the equivalence; see Corollary 1 given later. Note also that finding an appropriate context $E$ may not be easy in general; we discuss how to mechanically find $E$ in Section 5.

*Example 3.* Recall again formula (2) from Section 2.3. Let us define $C, D$, and $E$ by:

$$C \stackrel{\triangle}{=} \lambda(x,y).\exists r.[\,](x,y,r)$$
$$E \stackrel{\triangle}{=} \lambda(x,y).y=0 \lor (y \neq 0 \land [\,](x,y-1))$$
$$D \stackrel{\triangle}{=} \lambda(x,y,r).(y=0 \land r=0) \lor \exists s.(y \neq 0 \land r = x+s \land [\,](x,y-1,s)).$$

Then, for any ternary predicate $X$, we have:

$$\begin{aligned} C[D[X]] &\equiv \lambda(x,y).\exists r.(y=0 \land r=0) \lor \exists s.(y \neq 0 \land r = x+s \land X(x,y-1,s)) \\ &\equiv \lambda(x,y).y=0 \lor \exists r,s.(y \neq 0 \land r = x+s \land X(x,y-1,s)) \\ &\equiv \lambda(x,y).y=0 \lor (y \neq 0 \land \exists s.X(x,y-1,s)) \equiv E[C[X]]. \end{aligned}$$

By Theorem 1, we have $C[D[\mathit{Mult}]] \sqsupseteq \mu Y(x,y).y=0 \lor (y \neq 0 \land Y(x,y))$. Thus, the goal $\forall x,y.y<0 \lor \exists r.\mathit{Mult}(x,y,r)$ has been reduced to:

$$\forall x,y.y<0 \lor (\mu Y(x,y).y=0 \lor (y \neq 0 \land Y(x,y)))(x,y),$$

which can be proved valid by `Mu2CHC`. □

#### 3.3 Fold/Unfold for $\nu$-Formulas

We now prove a theorem that allows us to replace a predicate of the form $C[\nu X.D[X]]$ with one of the form $\nu Y.E[Y]$. It is similar to Theorem 1, but requires more conditions. Recall Lemma 1, which provides a sufficient syntactic condition for the co-continuity.

**Theorem 2.** *Let $C, D$ and $E$ be $(k,\ell)$, $(k,k)$, and $(\ell,\ell)$-contexts respectively. Suppose that the following conditions hold: (i) $C[\top^{(k)}] \sqsupseteq_\ell \top^{(\ell)}$, (ii) $C[D[X]] \sqsupseteq_\ell E[C[X]]$, and (iii) $C$ is co-continuous. Then $C[\nu X(x_1,\dots,x_k).D[X](x_1,\dots,x_k)] \sqsupseteq \nu Y(y_1,\dots,y_\ell).E[Y](y_1,\dots,y_\ell)$.*

*Proof.* For $F \in \mathbf{D}_k \to \mathbf{D}_k, f \in \mathbf{D}_k$ and an ordinal $\gamma$, we define $F^\gamma(\top^{(k)})$ inductively by: $F^0(\top^{(k)}) = \top^{(k)}$, $F^{\gamma+1}(\top^{(k)}) = F(F^\gamma(\top^{(k)}))$, and $F^\gamma(\top^{(k)}) = \sqcap_{\gamma'<\gamma} F^{\gamma'}(\top^{(k)})$ if $\gamma$ is a limit ordinal. By abuse of notation, we write $D^\gamma[\top^{(k)}]$ for $[\![D]\!]^\gamma(\top^{(k)})$ if $D$ is a $(k,k)$-context. Since there exists an ordinal $\gamma$ such that $\nu X.D[X] = D^\gamma[\top^{(k)}]$ and $\nu Y.E[Y] = E^\gamma[\top^{(\ell)}]$, it suffices to show that $C[D^\gamma[\top^{(k)}]] \sqsupseteq_\ell E^\gamma[\top^{(\ell)}]$ holds for any ordinal $\gamma$, by transfinite induction on $\gamma$. The base case where $\gamma = 0$ follows immediately from the first condition. If $\gamma$ is a successor ordinal $\gamma'+1$, then

$$C[D^\gamma[\top]] \sqsupseteq E[C[D^{\gamma'}[\top]]] \sqsupseteq E[E^{\gamma'}[\top]] \equiv E^\gamma[\top].$$

Here, we have used the induction hypothesis in the second inequality. If $\gamma$ is a limit ordinal, then we have:

$$C[D^\gamma[\top]] \equiv C[\sqcap_{\gamma'<\gamma}(D^{\gamma'}[\top])] \equiv \sqcap_{\gamma'<\gamma} C[D^{\gamma'}[\top]] \sqsupseteq \sqcap_{\gamma'<\gamma} E^{\gamma'}[\top] \equiv E^\gamma[\top].$$

Here we have used the co-continuity in the second inequality. We have thus proved $C[D^\gamma[\top^{(k)}]] \sqsupseteq_\ell E^\gamma[\top^{(\ell)}]$ holds for any ordinal $\gamma$. We, therefore, have $C[\nu X(\widetilde{x}).D[X](\widetilde{x})] \sqsupseteq \nu Y(\widetilde{y}).E[Y](\widetilde{y})$ as required. □

*Example 4.* Recall the formula $\forall x, y, a, r.\overline{Mult}(x, y, r) \vee Multacc(x, y, a, r + a)$ discussed in Section 3.1. Let us define $C, D, E$ by:

$$
\begin{aligned}
C &\stackrel{\triangle}{=} \lambda(x, y, a, r).[\,](x, y, r) \vee Multacc(x, y, a, r + a)\\
D &\stackrel{\triangle}{=} \lambda(x, y, r).((y \neq 0 \vee r \neq 0) \wedge \forall s.(y = 0 \vee r \neq x + s \vee [\,](x, y - 1, s)))\\
E &\stackrel{\triangle}{=} \lambda(x, y, a, r).y = 0 \vee [\,](x, y - 1, x + a, r - x)
\end{aligned}
$$

They satisfy all the three conditions of Theorem 2. In particular, for any ternary predicate $X$, we have

$$
\begin{aligned}
C[D[X]] &\equiv \lambda(x, y, a, r).((y \neq 0 \vee r \neq 0)\wedge\\
&\qquad \forall s.(y = 0 \vee r \neq x + s \vee X(x, y - 1, s))) \vee Multacc(x, y, a, r + a)\\
&\equiv \lambda(x, y, a, r).((y \neq 0 \vee r \neq 0)\wedge\\
&\qquad \forall s.(y = 0 \vee r \neq x + s \vee X(x, y - 1, s)))\\
&\qquad\quad \vee(y = 0 \wedge r + a = a) \vee (y \neq 0 \wedge Multacc(x, y - 1, x + a, r + a))\\
&\equiv \lambda(x, y, a, r).y = 0 \vee X(x, y - 1, r - x)\vee\\
&\qquad Multacc(x, y - 1, x + a, r + a))\\
&\equiv E[C[X]],
\end{aligned}
$$

based on the corresponding transformations shown in Section 3.1. We have thus $\forall x, y, a, r.\overline{Mult}(x, y, r) \vee Multacc(x, y, a, r + a) \sqsupseteq \forall x, y, a, r.(\nu Y(x, y, a, r).y = 0 \vee Y(x, y - 1, x + a, r - x))(x, y, a, r)$, and the righthand side can be proved to be valid by `Mu2CHC`. □

Note that Theorems 1 and 2 guarantee the soundness of the replacement of $C[\alpha X(x_1, \ldots, x_k).D[X](x_1, \ldots, x_k)]$ with $\nu Y(y_1, \ldots, y_\ell).E[X](y_1, \ldots, y_\ell)$ (for $\alpha \in \{\mu, \nu\}$), but not completeness: the validity of $C[\alpha X(x_1, \ldots, x_k).D[X](x_1, \ldots, x_k)]$ does not necessarily imply that of $\nu Y(y_1, \ldots, y_\ell).E[X](y_1, \ldots, y_\ell)$. Actually, by combining Theorem 1 and the dual version of Theorem 2, we obtain the following corollary, which guarantees completeness under a stronger condition.

**Corollary 1.** *Let $C, D$ and $E$ be $(k, \ell)$, $(k, k)$, and $(\ell, \ell)$-contexts respectively. Suppose that the following conditions hold: (i) $C[\bot^{(k)}] \sqsubseteq_\ell \bot^{(\ell)}$, (ii) $C[D[X]] \equiv_\ell E[C[X]]$, and (iii) $C$ is continuous. Then $C[\mu X(x_1, \ldots, x_k).D[X](x_1, \ldots, x_k)] \equiv_\ell \mu Y(y_1, \ldots, y_\ell).E[Y](y_1, \ldots, y_\ell)$.*

### 4 Further Examples

In this section, we give more examples to demonstrate the utility of our transformations for relational/temporal property verification of recursive programs.

#### 4.1 Relational Reasoning on Recursive Programs

Below we discuss an example which is beyond the reach for state-of-the-art CHC solvers (see e.g., [33], the end of Section 5).

*Example 5.* Consider the goal $\forall x, y, z, r.(\mathit{Mult}(x+y, z, r) \Rightarrow \exists s, t.\mathit{Mult}(x, z, s) \wedge \mathit{Mult}(y, z, t) \wedge r = s + t)$, which is equivalent to:

$$\forall x, y, z, r.(\overline{\mathit{Mult}}(x+y, z, r) \vee \exists s, t.(\mathit{Mult}(x, z, s) \wedge \mathit{Mult}(y, z, t) \wedge r = s + t)),$$

where $\mathit{Mult}$ and $\overline{\mathit{Mult}}$ are as given in Section 2.3. The following contexts $C, D$, and $E$ satisfy the following three conditions of Theorem 2.

$$C \triangleq \lambda(x, y, z, r).[\,](x+y, z, r) \vee \exists s, t.(\mathit{Mult}(x, z, s) \wedge \mathit{Mult}(y, z, t) \wedge r = s + t)$$
$$D \triangleq \lambda(x, z, r).(z \neq 0 \vee r \neq 0) \wedge (z = 0 \vee [\,](x, z-1, r-x))$$
$$E \triangleq \lambda(x, y, z, r).(z = 0 \vee (z \neq 0 \wedge [\,](x, y, z-1, r-x-y))).$$

By Theorem 2, we have $C[\overline{\mathit{Mult}}] \sqsupseteq \nu Y(x, y, z, r).E[Y](x, y, z, r) \equiv \lambda(x, y, z, r).\top$. We have thus proved that $\forall x, y, z, r.C[\overline{\mathit{Mult}}](x, y, z, r)$ (i.e., $\forall x, y, z, r.(\mathit{Mult}(x+y, z, r) \Rightarrow \exists s, t.\mathit{Mult}(x, z, s) \wedge \mathit{Mult}(y, z, t) \wedge r = s + t)$) is valid. □

### 4.2 Proving Temporal Properties

Here we give an example of proving a liveness property of a recursive program by using our transformation. The example is a variation of the example discussed in [22], but it cannot be handled by their method for proving MuArith formulas.

*Example 6.* Consider the following OCaml program:

```
let rec sum n = if n=0 then 0 else n+sum(n-1)
let rec loop x = if x=0 then () else loop (x-1)
let rec repeat n = let x = sum n in loop x; repeat(n+1)
let main() = repeat 0
```

Suppose that we wish to prove that the function `repeat` is called infinitely often. The reduction from linear-time temporal property verification to MuArith yields the problem of determining the validity of $\mathit{Repeat}(0)$, where:

$$\mathit{Repeat} \triangleq \nu\mathit{Repeat}(n).(\exists x.\mathit{Sum}(n, x)) \wedge (\forall x.\overline{\mathit{Sum}}(n, x) \vee \mathit{Loop}(x)) \wedge \mathit{Repeat}(n+1)$$
$$\mathit{Sum} \triangleq \mu\mathit{Sum}(n, x).(n = 0 \wedge x = 0) \vee (n \neq 0 \wedge \exists r.\mathit{Sum}(n-1, r) \wedge x = n + r)$$
$$\mathit{Loop} \triangleq \mu\mathit{Loop}(x).x = 0 \vee (x \neq 0 \wedge \mathit{Loop}(x-1)).$$

Here, $\overline{\mathit{Sum}}$ is the De Morgan dual of $\mathit{Sum}$. The validity of this formula cannot be proved by Mu2CHC due to the existential quantifier. Note that Mu2CHC replaces each existential quantifier $\exists x.\varphi$ with a bounded quantifier $\exists x \leq a.\varphi$, and $a$ must be a linear expression. In the example above, $x$ is not linearly bounded by $n$. To remove the existential quantifier, let

$$C \triangleq \lambda n.\exists x.[\,](n, x)$$
$$E \triangleq \lambda n.n = 0 \vee (n \neq 0 \wedge [\,](n-1))$$
$$D \triangleq \lambda(n, x).(n = 0 \wedge x = 0) \vee (x \neq 0 \wedge \exists r.[\,](n-1, r) \wedge x = n + r).$$

**Algorithm 1:** Fold/unfold for disjunction

**Input:** Formula $\Phi$ of the form $X(f(x,y)) \vee Y(g(x,y))$, where $X$ and $Y$ are predicates defined by $\alpha_X, \alpha_Y \in \{\mu, \nu\}$.
**Output:** A formula $\Phi'$ such that $\Phi \sqsupseteq \Phi'$.

$\alpha \leftarrow$ if $\nu \in \{\alpha_X, \alpha_Y\}$ then $\nu$ else $\mu$;
$\bigwedge \psi_i \leftarrow$ `cnf(unfold(`$\Phi$`))`;
**for each** $\psi_i$ **do**
  **if** $\psi_i$ has the form $X(s_1) \vee Y(s_2) \vee \psi'_i$, $f(t_1,t_2) \equiv s_1$ and $g(t_1,t_2) \equiv s_2$ **then**
    $\psi_i \leftarrow Z(t_1,t_2) \vee \psi'_i$;
**return** $\alpha Z(x,y). \bigwedge \psi_i$;

Since $C[D[X]] \sqsupseteq E[C[X]]$ holds, we can apply Theorem 1 to underapproximate $\exists x.Sum(n,x)$ by $\mu X(n).n = 0 \vee (n \neq 0 \wedge X(n-1))$. Therefore, the goal has been reduced to $Repeat'(0)$ where

$$\begin{aligned} Repeat' &\stackrel{\triangle}{=} \nu Repeat'(n).X(n) \wedge (\forall x.\overline{Sum}(n,x) \vee Loop(x)) \wedge Repeat'(n+1) \\ X &\stackrel{\triangle}{=} \mu X(n).n = 0 \vee (n \neq 0 \wedge X(n-1)), \end{aligned}$$

which can be proved valid by `Mu2CHC` automatically. □

### 5 Algorithm and Evaluation

In this section, we first present an algorithm for our transformation and then outline its implementation and report on experimental results.

#### 5.1 Algorithm

Theorems 1 and 2 given in Section 3 state sufficient conditions for our fold/unfold transformation to be sound. In this subsection, we discuss how to systematically apply the theorems and how to find a context $E$.

To make it easy to find $E$, we restrict input formulas of our transformations to those of the form $X(f(x,y)) \vee Y(g(x,y))$, $X(f(x,y)) \wedge Y(g(x,y))$, and $\exists y.X(f(x,y))$, where $X$ and $Y$ are predicates defined by fixpoint operators, and $f(x,y)$ and $g(x,y)$ denote (possibly sequences of) terms that may contain free variables $x$ and $y$. For the sake of simplicity, we assume here that the definitions for $X$ and $Y$ are independent; $X$ cannot be obtained by unfolding $Y$, and vice versa. Transformations for more complex formulas like the one in Example 5 can be achieved by repeatedly applying the transformations for smaller contexts.

The transformation algorithm for disjunctive formulas is shown in Algorithm 1. It takes as input a formula $\Phi = X(f(x,y)) \vee Y(g(x,y))$ and outputs an underapproximation $\Phi'$ of $\Phi$. It can take $[\,](f(x,y)) \vee Y(g(x,y))$ or $X(f(x,y)) \vee [\,](g(x,y))$ as the context $C$ and apply Theorem 2 if $X$ or $Y$ is

**Algorithm 2:** Fold/unfold for $\exists$

**Input:** Formula $\Phi$ of the form $\exists y.X(f(x,y))$, where $X$ is a predicate defined by $\mu$ or $\nu$.

**Output:** A formula $\Phi'$ such that $\Phi \sqsupseteq \Phi'$.

1 $\bigvee \psi_i \leftarrow$ `dnf(normalize`$_\exists$`(unfold(`$\Phi$`)))`;

2 **for each** $\psi_i$ **do**

3     **if** $\psi_i$ has the form $(\exists z.X(s)) \wedge \psi_i'$, and $f(t_x, y) \equiv [t_z/z]s$, where $\mathbf{FV}(t_x) \subseteq \{x\}, \mathbf{FV}(t_z) \subseteq \{x, y\}$ **then**

4         $\psi_i \leftarrow Z(t_x) \vee \psi_i'$;

5 **return** $\mu Z(x). \bigvee \psi_i$; defined by $\nu$, and Theorem 1 otherwise (line 1). On line 2, the algorithm unfolds $X$ and $Y$[6] and then normalizes the resulting formula to a conjunctive normal form (CNF), where quantified formulas are treated as atomic. It then applies the "fold" transformation to each conjunct $\psi_i$. To this end, for each $\psi_i$ that contains $X(s_1) \vee Y(s_2)$, the algorithm finds terms $t_1$ and $t_2$ such that $X(s_1) \vee Y(s_2) \equiv X(f(t_1, t_2)) \vee Y(g(t_1, t_2))$; this is achieved by solving the unification constraints $s_1 \equiv f(x', y')$ and $s_2 \equiv g(x', y')$ modulo arithmetic theories, where $x'$ and $y'$ are treated as variables but $x$ and $y$ are treated as constants. Finally, the algorithm replaces $X(s_1) \vee Y(s_2)$ with $Z(t_1, t_2)$, where $Z(x, y)$ is a new predicate that corresponds to $X(f(x, y)) \vee Y(g(x, y))$.

We omit the transformation algorithm for conjunctive formulas since it is similar to the case above, except that the new predicate $Z$ is bound by $\mu$ (note that condition (i) of Theorem 2 may not be satisfied), and that it converts the unfolded formula to a disjunctive normal form (DNF), instead of CNF.

The algorithm for existential formulas is shown in Algorithm 2. It unfolds $X$, normalizes existential quantifiers, and obtains a DNF. In the normalization of existential quantifiers, it moves existential quantifiers inwards (by using, e.g., the law $\exists x.(\psi_1 \vee \psi_2) \equiv (\exists x.\psi_1) \vee (\exists x.\psi_2)$) and eliminates them as much as possible (by using, e.g., the equality-based quantifier elimination). For each disjunct $\psi_i$ of the form $(\exists z.X(s)) \wedge \psi_i'$, it finds $t_x$ and $t_z$, such that $f(t_x, y) \equiv [t_z/z]s$ (again, by performing unification modulo arithmetic theories), and replaces the disjunct with $Z(t_x) \wedge \psi_i'$. Here, $Z(t_x)$ corresponds to $\exists y.X(f(t_x, y))$, and $t_z$ serves as a witness for $X(f(t_x, y)) \Rightarrow \exists z.X(s)$.

#### 5.2 Implementation and Experiments

We have implemented the transformation in a tool called `MuFolder` based on the algorithms discussed above, on top of the `AdtInd` theorem prover [37], using its routines for pattern-matching, normalization, and simplification. For the implication checks, `MUnfold` uses the `Z3` SMT solver [27]. `MuFolder` can be tested at https://www.kb.is.s.u-tokyo.ac.jp/~koba/mu/.

[6] If none of $\psi_i$'s are changed in the loop on lines 3-5, we may backtrack and unfold $X$ and $Y$ more than once.

**Table 1.** Experiments.

| # | input formula $\Phi$ | output formula $\Phi'$ |
|---|---|---|
| 1 | $\overline{Even}(x) \vee Odd(x+1)$ | $\nu Z(x).x = 0 \vee Z(x-2)$ |
| 2 | $\overline{Even}(x) \vee \overline{Odd}(x)$ | $\nu Z(x).(x \neq 0 \vee \overline{Even}(x-1)) \wedge Z(x-1)$ |
| 3 | $Even(x) \vee \overline{Odd}(x+1)$ | $\nu Z(x).x = 0 \vee Z(x-2)$ |
| 4 | $\overline{Mult}(x+y,z,r) \vee \exists s.Mult(x,z,s)$ | $\nu Z(x,y,z,r).z = 0 \vee Z(x,y,z-1,r-(x+y))$ |
| 5 | $\overline{Mult}(x+y,z,r) \vee \exists s_1,s_2.$ $Mult(x,z,s_1) \wedge Mult(y,z,s_2) \wedge r = s_1 + s_2$ | $\nu Z(x,y,z,r).z = 0 \vee Z(x,y,z-1,r-(x+y))$ |
| 6 | $\overline{Mult}(2x+3y,z,r) \vee \exists s_1,s_2.$ $Mult(x,z,s_1) \wedge Mult(y,z,s_2) \wedge r = 2s_1 + 3s_2$ | $\nu Z(x,y,z,r).z = 0 \vee$ $z \neq 0 \wedge Z(x,y,z-1,r-(2x+3y))$ |
| 7 | $\overline{Mult}(x,y,r) \vee Mult(x,y,r)$ | $\nu Z(x,y,r).y = 0 \vee y \neq 0 \wedge Z(x,y-1,r-x)$ |
| 8 | $\overline{Mult}(x,y,r) \vee MultAcc(x,y,a,r+a)$ | $\nu Z(x,y,a,r).y = 0 \vee$ $y \neq 0 \wedge Z(x,y-1,x+a,r-x)$ |
| 9 | $\exists r.Mult(x,y,r)$ | $\mu Z(x,y).y = 0 \vee y \neq 0 \wedge Z(x,y-1)$ |
| 10 | $\overline{Plus}(x+y,z,r) \vee \exists s.Plus(x,z,s)$ | $\nu Z(x,y,z,r).z = 0 \vee Z(x,y,z-1,r-1)$ |
| 11 | $\overline{Plus}(4x-3y,z,r) \vee \exists s_1,s_2.$ $Plus(x,z,s_1) \wedge Plus(y,z,s_2) \wedge r = 4s_1 - 3s_2$ | $\nu Z(x,y,z,r).z = 0 \vee Z(x,y,z-1,r-1)$ |
| 12 | $\exists r.Sum(x,r)$ | $\mu Z(x).x = 0 \vee x \neq 0 \wedge Z(x-1)$ |

We have evaluated `MuFolder` on several benchmarks outlined in Table 1. These benchmarks include formulas obtained from the relational and temporal verification properties; some of which have been taken from the benchmark set for Unno et al.'s induction-based CHC solver [33] and modified to include both $\mu$ and $\nu$. We have confirmed that all the benchmark problems can be solved in our approach within a few seconds. To our knowledge, except the formulas 7, 8 (for which the method of [33] can be used) and 10,11 (for which `Mu2CHC` works), `Mu2CHC` (without our transformation) or the existing CHC solvers cannot directly prove the validity of the formulas. Note that formula 12 comes from Example 6. The combination of the transformation with `Mu2CHC` enables fully automated verification of Example 6.

### 6 Related Work

As already mentioned, fold/unfold transformations have been originally proposed for logic programming [32], and later extended for CHC (a.k.a. constraint logic programs) [1,17]. Those transformations have originally been proposed to speed up program execution, but recently, Mordvinov and Fedyukovich [26] and De Angelis et al. [15] shown that related transformations are also useful in the context of verification based on CHC solving. Those transformations correspond to the transformation for the $\nu$-only fragment of `MuArith`.[7] Our transformation can thus be considered an extension of fold/unfold-like transformations to `MuArith`, which allows alternations of least/greatest fixpoints. Sato [31] studied an extension of fold/unfold transformations for a first-order logic, where negations and quantifiers are allowed in clause bodies; thus, some mixtures of least/greatest fixpoints are allowed. The correctness of his transformation is, however, based on a three-valued logic, hence different from `MuArith`. The correctness of most of the

[7] This is because, although the semantics of each predicate is interpreted as the least fixpoint, the predicates occur in negative positions in goal clauses.

transformations mentioned above is guaranteed by some syntactic conditions, while our transformation is based on semantic conditions.

Unno et al. [33] proposed a method for automatically solving CHC problems by using induction. Their method is based on a tailor-made proof system; hence it is difficult to integrate the method with other CHC or `MuArith` solvers (in fact, that disadvantage motivated the above-mentioned work of De Angelis et al. [15]). Their method slightly goes beyond the CHC satisfiability (or the $\nu$-only fragment of `MuArith`) but cannot deal with complex combinations of least/greatest fixpoints and quantifiers (like $\forall x, y, z, r.(Mult(x+y, z, r) \Rightarrow \exists s, t.Mult(x, z, s) \wedge Mult(y, z, t) \wedge r = s + t)$, discussed in Section 4).

As mentioned in Section 1, fixpoint logic-based approaches to program verification (including CHC-based ones) have been drawing attention. Kobayashi et al. [22,23,35] have shown that temporal property verification of (higher-order) programs can be reduced to the validity checking of (higher-order) fixpoint logic formulas. They proposed a concrete method for checking validity of first-order fixpoint formulas and implemented a validity checking tool `Mu2CHC`. As discussed already, our transformations can be used to improve the capability of `Mu2CHC`. Another thread of work on a fixpoint logic-based approach to system verification is that of Parameterized Boolean Equation Systems (PBES) [21]. Actually, `MuArith` may be considered an instance of PBES, where data are restricted to integers. Groote, Willemse, and others [10,14,21,30,36] studied applications of PBES to verification of infinite state systems, and devised various techniques for solving PBES. To our knowledge, however, they have not studied fold/unfold transformations for PBES.

### 7 Conclusions

We have formalized fold/unfold-like transformations for a fixpoint logic, and shown that they are useful for verification of relational/temporal properties of recursive programs. We have implemented the transformations, and shown their effectiveness through experiments.

**Acknowledgments.** We would like to thank anonymous referees for useful comments, especially for bringing the work on PBES to our attention. This work was supported in part by the University of Tokyo-Princeton Strategic Partnership Grant, JSPS KAKENHI Grant Number JP15H05706, and NSF (USA) award FMitF 1837030.
